# Round the Hospitals

**Published:** 1916-10-14

**Authors:** 


### ROUND THE HOSPITALS.

Now that the autumn session of Parliament is in being speculation is rife as to the prospects of the Registration Bill. We understand that at the meeting of the Council of the College of Nursing on Thursday week a communication was received from the Secretaries of the Central Committee for the State Registration of Nurses, criticising certain points in the draft Bill approved by the Council; to this a reply has been made by Mr. Stanley, giving arguments in support of the position taken up by the Council. It will be remembered that Mr. Stanley has repeatedly stated that he is hopeful of getting a measure for the State Registration of Nurses passed, even during the war, providing its sponsors can go to Parliament with 10,000 names on the first Register. It therefore rests with the nursing profession itself to see to it that this essential to success is provided. For many months now the scheme of the College of Nursing, its constitution and objects have been before trained nurses, who have had ample time and opportunity to realise the advantages of membership of such a body, an assurance to which recent events have given point; and the time has come for every trained and certificated nurse to make a decision as to her course, which in her own interests can only be to become a member of the College without delay. Applications to register are being received daily by the Secretary, Miss Rundle, at 6 Vere Street, Cavendish Square, London, W., who, with her staff, is dealing with them as expeditiously as possible. The Register of Members now being formed by the College is to be the first Register under the proposed Registration Act.

We are glad to have it on independent authority that the friction which used to prevail at the outset in the home provision for the treatment of our warriors in this country between commandant, matron, and their staff has now almost everywhere disappeared. It is one more instance of the good old English policy of mutual consideration, the spirit of compromise, and the recognition that in large establishments friendly co-operation between all officials and their staffs is essential to the success and the comfort of everybody concerned. Time has demonstrated that it matters very little to the commandant what the chief member of the nursing staff is called. All that he wishes to secure is efficiency in the nursing, and so long as that prevails, as it must prevail where the matron or sister-in-charge or head of the nursing is really efficient, friction disappears and peace and efficiency prevail. In these circumstances we shall hope to see that the nursing papers secure material to fill their space having an initial freshness and sound basis of fact.

The destruction by fire of the Civil Hospital at Rheims, reported recently in the press, will be a great loss to the city, and will be regretted by all who care for the advancement of nursing education in France. The hospital, a beautiful old religious house, afforded accommodation for 600 patients, and, besides being a Medical School of some repute, was one of the very few training-schools for nurses in France. The matron, Mlle. Luigi, who was trained at the London Hospital and knew Miss Cavell there, has for some years been doing valuable educational work amongst her countrywomen, adapting the English system of lectures, classes, besides instruction, etc., to the needs of French probationers; the nurses receive a certificate of training after two years of continuous ward work. It was undoubtedly due to Mlle. Luigi's efforts that the sanitary arrangements at this hospital were roomy and convenient, while the presence of dainty covers, flowers, etc., made the large ancient wards strangely like those of an English hospital. The destruction of this hospital was probably due to its close proximity to the famous and beautiful Church of St. Remi, which has for many months been the target for German incendiary shells, but has so far escaped serious injury. Mlle. Luigi and her brave staff had been working quietly on amidst falling shells for many months. Some of them have lost their lives, and the matron has herself had many narrow escapes, but it was necessary that an institution where women and children injured in the bombardment could be cared for should remain open, and so the work went on. Wards when wrecked were immediately repaired, and the nurses were able, by speedily removing the patients to underground wards, to avoid some of the risks during seven bombardments. It will be remembered that Rheims has been occupied by the German armies, and it speaks much for Mlle. Luigi's tact and *savoir faire* that on the occasion when the hospital was overrun by the Hun soldiers she was able to protect her nurses from any sort of insult.

For Matrons' and Sisters' Appointments, see p. vi.

